# Durvalumab induced sarcoid‐like pulmonary lymphadenopathy

**DOI:** 10.1002/rcr2.542

**Published:** 2020-02-25

**Authors:** Emma Sanderson, Hari Wimaleswaran, Clare Senko, Shane White, Christine F. McDonald

**Affiliations:** ^1^ Department of Respiratory and Sleep Medicine Austin Health Melbourne VIC Australia; ^2^ Faculty of Medicine, Dentistry and Health Sciences University of Melbourne Melbourne VIC Australia; ^3^ Department of Medical Oncology Austin Health Melbourne VIC Australia

**Keywords:** Durvalumab, immune checkpoint inhibition, sarcoidosis

## Abstract

Immune checkpoint inhibitors (ICIs) have become pivotal in the treatment of lung cancer. An increasing number of immune‐related adverse events (irAEs) have been recognized with their use. To our knowledge, this is the first published case of sarcoid‐like pulmonary lymphadenopathy associated with durvalumab, a monoclonal antibody against programmed death ligand‐1 (PD‐L1). A 76‐year‐old woman received adjuvant durvalumab for Stage IIA pT2aN1M0 (American Joint Committee on Cancer, Seventh edition) poorly differentiated lung adenocarcinoma. After three cycles, a sarcoid‐like granulomatous reaction was identified in mediastinal and hilar lymph nodes. Although the lymphadenopathy remained stable in size with the ongoing treatment, progressive intracranial metastases were identified after a further three cycles of durvalumab. Sarcoid‐like inflammation with the formation of non‐caseating granulomas in the absence of systemic sarcoidosis is an irAE which may mimic disease progression. Although a subset of patients who experience this reaction may have a favourable response to checkpoint inhibition, progression of disease may occur contemporaneously.

## Graphical abstract

Immune checkpoint inhibitors, such as durvalumab, are associated with a variety of unique immune‐related adverse events. In this case, sarcoid‐like mediastinal and hilar lymphadenopathy developed with the use of adjuvant durvalumab for the treatment of lung adenocarcinoma.

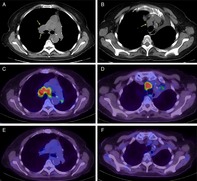

## Introduction

Immune checkpoint inhibitors (ICIs) are monoclonal antibodies that enhance anti‐tumour immunity by targeting molecules which downregulate T‐cell responses 1.

Immune‐related adverse events (irAE) are toxicities unique to checkpoint blockade and may affect any organ system with varying severity. With the widespread use of ICIs, there has been increased appreciation of rheumatological irAEs 1. Many of these share a similar phenotype to those documented in the general population such as systemic lupus erythematous, polymyalgia rheumatica, and sarcoidosis 1.

Sarcoidosis is a granulomatous disease characterized by the formation of non‐caseating granulomas in multiple organ systems 2. Sarcoid‐like granulomatous inflammation is an uncommon irAE that has been associated with inhibition of cytotoxic T lymphocyte antigen‐4 (CTLA‐4) with ipilimumab, programmed death‐1 (PD‐1) with nivolumab and pembrolizumab, and PD‐L1 with atezolizumab and avelumab 2, 3, 4. We report a case of sarcoid‐like granulomatous lymphadenopathy associated with durvalumab, an anti‐PD‐L1 selective human immunoglobulin G1 (IgG1) monoclonal antibody in a woman with lung adenocarcinoma.

## Case Report

**Figure 1:**
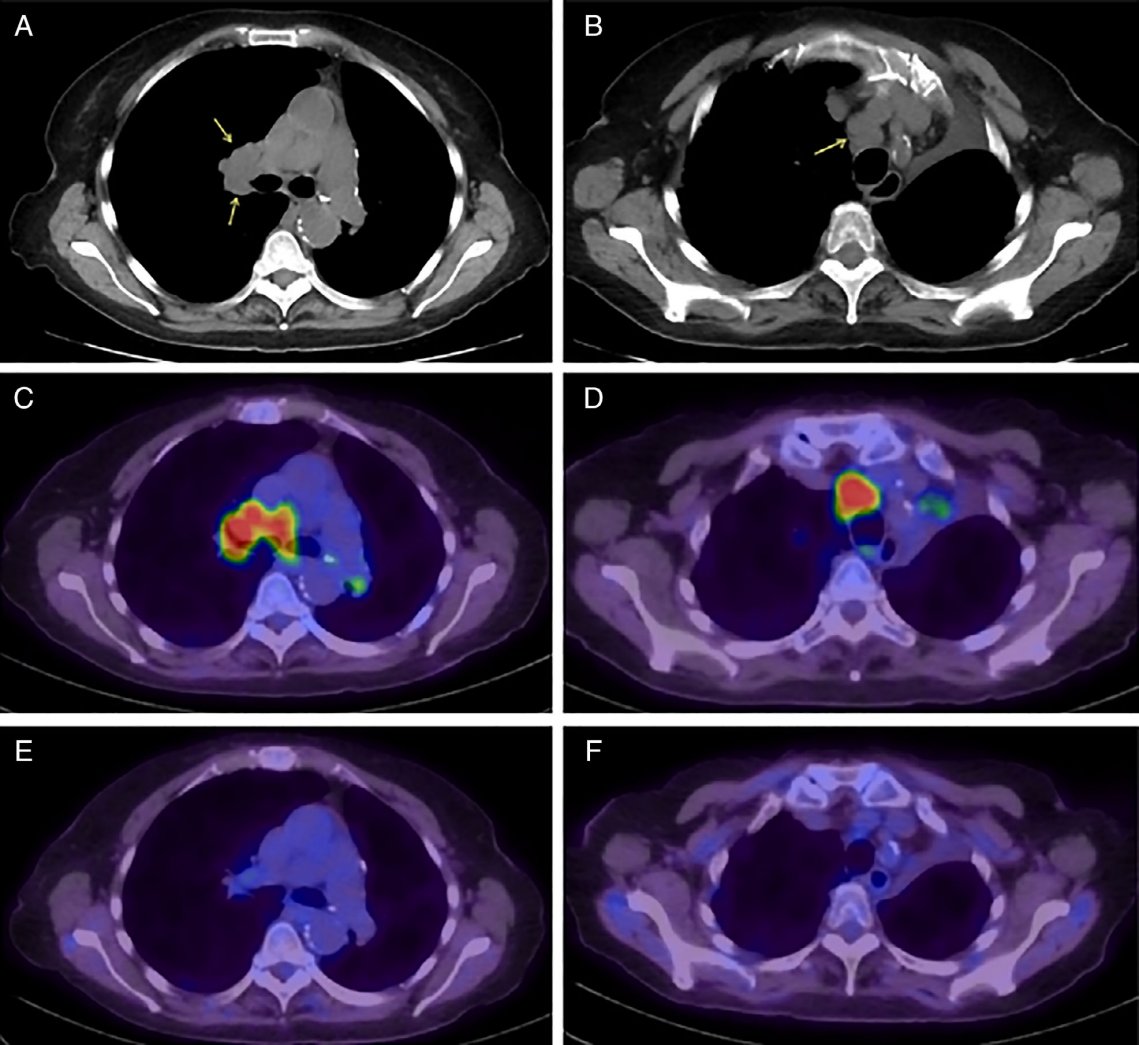
(A, B) Non‐contrast computed tomography (CT) of the chest with bilateral mediastinal lymphadenopathy involving the right pre‐tracheal nodal station, left pre‐vascular space, and subcarina. (C, D) Positron emission tomography (PET)‐CT demonstrating highly metabolic active lymphadenopathy. (E, F) PET‐CT three months after the cessation of durvalumab demonstrating complete resolution of metabolically active bilateral hilar and mediastinal lymphadenopathy.

A 76‐year‐old woman was diagnosed with a Stage IIA (pT2aN1M0) poorly differentiated lung adenocarcinoma after presenting with haemoptysis and shoulder pain. Whole‐body positron emission tomography‐computed tomography (PET‐CT) identified a spiculated 4.5 cm 18F‐fluorodeoxyglucose (FDG)‐avid lung mass in the apicoposterior segment of the left upper lobe without evidence of nodal or distant metastases. The patient underwent a left upper lobe lobectomy and completed four cycles of adjuvant chemotherapy (cisplatin and vinorelbine) in January 2019. The specimen was KRAS mutant, ALK negative and EGFR/BRAF wild type with clear margins. One hilar lymph node was affected with metastatic disease. Prominent anthracosilicosis with non‐necrotizing granulomatous inflammation was noted in most lymph nodes sampled (Fig. 1).

Past medical history included hypertension, depression, thyrotoxicosis, and hysterectomy. The patient was an ex‐tobacco smoker with a 40 pack‐year‐history.

The patient commenced the first cycle of adjuvant durvalumab (20 mg/kg every four weeks for 12 months) on 25 February 2019.

Routine whole‐body non‐contrast CT three months after the initiation of durvalumab was suspicious for nodal recurrence with interval development of mediastinal lymphadenopathy. PET confirmed highly metabolically active bilateral mediastinal and hilar lymphadenopathy in addition to areas of increased FDG‐uptake in the right scapula, left iliac crest, posterior ilium, and a 4‐mm right upper lobe lung nodule.

Fine needle aspirate (FNA) samples obtained via endobronchial ultrasound (EBUS) bronchoscopy (station 7, 4R, and 11R) demonstrated epithelioid histiocytes arranged in non‐caseating granulomas. Anthracotic pigment and flecks of polarizable silicotic material were identified in most of the granulomas. No malignant cells or fungi were identified and both smear and culture for acid‐fast bacilli were negative. A diagnosis of durvalumab‐associated sarcoid‐like lymphadenopathy was made. The patient was asymptomatic and durvalumab was continued with no delay to the treatment schedule.

**Figure 2:**
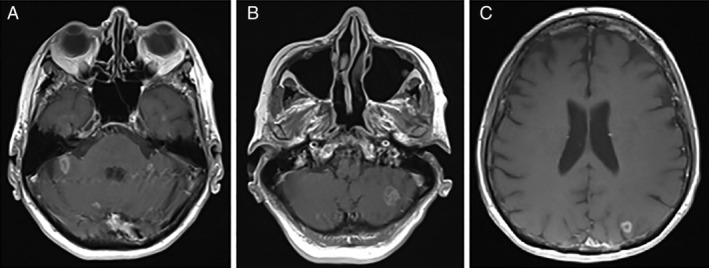
Axial T1‐weighted turbo‐spin echo (TSE) magnetic resonance imaging (MRI) demonstrating multifocal enhancing cerebellar (A, B) and cerebral lesions (C) with adjacent vasogenic oedema and no leptomeningeal involvement.

Repeat CT staging was completed after the sixth cycle of durvalumab in August 2019. Although there was no significant change in size of the mediastinal and hilar lymphadenopathy, a ring‐enhancing lesion suspicious for metastatic disease was identified in the right cerebellum. Subsequent magnetic resonance imaging (MRI) of the brain confirmed numerous intra‐axial ring‐enhancing lesions (Fig. 2).

Although the neuroimaging findings were considered most consistent with intracranial metastases rather than neurosarcoidosis, a two‐week trial of high‐dose prednisolone was commenced. MRI of the brain repeated following this trial did not show any significant changes to the intracranial lesions, supporting a diagnosis of intracranial metastases. Durvalumab was ceased and the patient was referred for whole‐brain radiotherapy. Oral prednisolone was continued at a dose of 25 mg daily. The dosage was weaned over the course of two months prior to cessation in October 2019.

Repeat PET‐CT in November, three months after the cessation of durvalumab, demonstrated complete resolution of the metabolically active bilateral hilar and mediastinal lymphadenopathy. In addition, there was resolution of the previously identified areas of increased FDG‐uptake in the right upper lobe, right scapula, and left ilium.

## Discussion

The development of sarcoid‐like granulomas with checkpoint inhibition may mimic disease progression and pose a clinical conundrum which may impact treatment decisions. The most common sites of involvement are the mediastinal and hilar lymph nodes (71% of cases), lung parenchyma (60% of cases), and skin (54.5% of cases) 2.

It is presumed that sarcoid‐like granulomas with ICIs occur via a similar mechanism to systemic sarcoidosis with a predominant type 1 T helper cell (Th1) response 2. The cell‐mediated immune response evoked by ICIs and subsequent destruction of immunogenic tumour cells may expose neoantigens which promote granulomatous inflammation 5.

Pulmonary involvement may manifest with a wide range of radiographic findings including bilateral patchy ground‐glass opacities, multifocal nodular changes, or focal consolidation with or without accompanying lymphadenopathy 2, 4.

Given that non‐necrotizing granulomatous inflammation was present in the lymph nodes sampled at the time of initial surgery, it is likely that there was a subclinical granulomatous process present prior to commencement of durvalumab. As these lymph nodes were not appreciated on initial PET‐CT and there are no prior images or histological specimens for review, we cannot comment on whether this process is related to her underlying malignancy or was long‐standing. The patient did not have features of systemic sarcoidosis at the time of presentation.

Despite the presence of non‐necrotizing granulomatous inflammation on the initial histological specimen, the commencement of durvalumab was temporally related with an increase in the size and degree of metabolic activity of the hilar and mediastinal lymphadenopathy on PET‐CT. This suggests that this process may have been accelerated by checkpoint inhibition.

As the patient received oral prednisolone for two months after durvalumab was ceased, it is unclear whether the resolution of lymphadenopathy on repeat PET‐CT was directly related to the cessation of checkpoint inhibition.

In this case, the appearance of intracranial metastatic disease occurred in the setting of stable mediastinal and hilar lymphadenopathy with ongoing administration of durvalumab. Although sarcoid‐like granulomas with ICIs have been associated with a favourable therapeutic response in a number of patients, this case is an example of disease recurrence occurring in close temporal proximity to biopsy‐proven sarcoid‐like granulomas, emphasizing the importance of close surveillance 5.

The differential diagnosis of neurosarcoidosis related to durvalumab was considered in this case. However, this rare manifestation was considered unlikely, given the absence of changes on MRI after the course of high‐dose corticosteroids and lack of leptomeningeal involvement. Further investigations such as lumbar puncture or meningeal biopsy were not undertaken.

Neurological involvement in systemic sarcoidosis and sarcoid‐like granulomas associated with ICIs most commonly affects the leptomeninges with evidence of leptomeningeal enhancement and thickening on MRI 6. However, MRI is not specific for neurosarcoidosis as there is a diverse range of radiographic appearances including parenchymal lesions which may mimic metastatic disease. These lesions are typically enhancing and closely associated with leptomeningeal involvement 7.

As with targeted therapies against CTLA‐4, PD‐1, and PD‐L1, our case has demonstrated an association between the anti‐PD‐L1 durvalumab and sarcoid‐like granulomatous lymphadenopathy.

In conclusion, in patients receiving ICIs, the development of sarcoid‐like granulomas and disease progression can occur contemporaneously. This case highlights the importance of ongoing surveillance for progressive or recurrent disease in this cohort. Further research is required to establish the natural history of this phenomenon and its relationship with tumour progression.

### Disclosure Statement

Appropriate written informed consent was obtained for publication of this case report and accompanying images.

## References

[1] Abdel‐Wahab N , Shah M , and Suarez‐Almazor ME . 2016 Adverse events associated with immune checkpoint blockade in patients with cancer: a systematic review of case reports. PLoS One 11(7):e0160221. 27472273 10.1371/journal.pone.0160221 PMC4966895

[2] Ramhia PH , Reichert B , Scott JF , et al. 2019 Immune checkpoint inhibitor‐induced sarcoidosis‐like granulomas. Int. J. Clin. Oncol. 24(10):1171–1181. 31321613 10.1007/s10147-019-01490-2

[3] Mitchell MA , Hogan K , and Amjadi K . 2018 Atezolizumab‐induced sarcoid‐like granulomatous reaction in a patient with urothelial cell carcinoma. Immunotherapy 10(14):1189–1192. 30326785 10.2217/imt-2018-0035

[4] Balestra R , Benzaquen S , and Wang J . 2017 Sarcoidosis‐like granulomatous lung reaction associated with anti–programmed death receptor‐1 ligand therapy. Ann. Am. Thorac. Soc. 14(2):296–299. 10.1513/AnnalsATS.201611-863LE 28146383

[5] Tetzlaff MT , Nelson KC , Diab A , et al. 2018 Granulomatous/sarcoid‐like lesions associated with checkpoint inhibitors: a marker of therapy response in a subset of melanoma patients. J. Immunother. Cancer 6(1):14. 29433571 10.1186/s40425-018-0323-0 PMC5810034

[6] Dunn‐Pirio AM , Shah S , and Eckstein C . 2018 Neurosarcoidosis following immune checkpoint inhibition. Case Rep. Oncol. 11(2):521–526. 30186134 10.1159/000491599 PMC6120397

[7] Ginat DT , Dhillon G , and Almast J . 2011 Magnetic resonance imaging of neurosarcoidosis. J. Clin. Imaging Sci. 1:15. 21977388 10.4103/2156-7514.76693 PMC3173834

